# Epidemiological Investigation of Goose Astrovirus in Hebei Province, China, 2019–2021

**DOI:** 10.3390/microorganisms12050990

**Published:** 2024-05-14

**Authors:** Ligong Chen, Huan Cui, Jiaqi Li, Yuxin Zhang, Heng Wang, Yejin Yang, Xuejing Wang, Cheng Zhang, Juxiang Liu

**Affiliations:** College of Veterinary Medicine, Hebei Agricultural University, Baoding 071000, China; clg01@163.com (L.C.); huancui1349@hebau.edu.cn (H.C.); ljq15027874688@163.com (J.L.); 15081207171@163.com (Y.Z.); wangheng946@163.com (H.W.); yejin317@outlook.com (Y.Y.); 13730474185@163.com (X.W.)

**Keywords:** goose astrovirus, epidemiological investigation, virus isolation, phylogenetic analysis

## Abstract

The goose astrovirus (GAstV), a key pathogen causing visceral gout and high mortality in geese, has spread widely in China, with frequent outbreaks in recent years. Outbreaks and transmissions of this virus have been reported across China, causing considerable economic losses to the goose industry worldwide, with losses exceeding tens of billions in China alone. However, there is still no effective prevention strategy against this virus. Therefore, continuous monitoring of the genetic diversity of dominant GAstV strains is crucial for developing targeted vaccines and appropriate therapeutics. As a crucial region for goose breeding in China, Hebei Province has previously lacked reports on the epidemiology of GAstV. Hence, investigating the epidemiology of GAstV in Hebei Province is highly important. From January 2019 to December 2021, 474 samples suspected of having a GAstV infection were collected in Hebei Province in this study. Through detailed histological observations, pathological examinations, virus isolation and identification, and genetic diversity analysis, we found that GAstV-2 has become the predominant circulating genotype. However, the presence of GAstV-1 and mixed infections cannot be ignored and should receive increased attention. The findings of this study not only deepened our understanding of GAstV in waterfowl in China but also provided scientific evidence for developing effective prevention and control measures, thereby promoting the healthy development of the goose industry in China.

## 1. Introduction

Astrovirus is a nonenveloped, positive-sense, and single-stranded RNA virus. The diameter of this type of virus is between 28 and 30 nm. Moreover, the surfaces of some virus particles exhibit unique five-pointed or six-pointed star shapes [1]. This type of virus mainly contains three open reading frames, namely, ORF1a, ORF1b, and ORF2. Among them, ORF1a and ORF1b encode nonstructural proteins that play a certain regulatory role in the virus replication process. ORF2 encodes the capsid protein, a protein whose primary role is to encapsulate viral nucleic acid and nucleic acid–protein complexes, which are critical to viral biology [2,3,4].

The International Committee on Taxonomy of Viruses (ICTV) has systematically classified the Astroviridae family into two major genera: Mamastroviruses (MAstVs) and Avastroviruses (AAstVs) [5,6,7]. The Mamastroviruses genus is further subdivided into six categories, encompassing astroviruses from humans, pigs, cats, minks, sheep, and dogs [8]. On the other hand, the Avastroviruses genus is classified into three types: AAstV-1, AAstV-2, and AAstV-3 [9]. Notably, AAstV-1 comprises only one member, turkey astrovirus type 1 (TAstV-1); AAstV-2 includes the nephritis virus (ANV) and chicken astrovirus (CAtsV); and AAstV-3 encompasses turkey astrovirus type 2 (TAstV-2), turkey astrovirus type 3 (TAstV-3), four distinct types of duck astroviruses (DAstV-1 to DAstV-4), and two goose astroviruses (GAstV-1 and GAstV-2) [10]. In addition to the abovementioned astroviruses, there are many other species of avian astroviruses. For example, astroviruses isolated from a variety of wild birds in tropical rainforests have not been formally classified, and the existence and characteristics of these viruses still need further study and classification [11,12].

Gout has become one of the key health issues faced by the global poultry industry, especially in goose breeding [1]. Gout is a complex metabolic disease of hyperuricemia that is closely related to primary and secondary kidney injury, and its pathogenesis is influenced by many factors. The etiology of gout is complex and diverse and often involves various factors related to feeding and management, such as nutritional imbalance, toxin exposure, and stress sources. It is also influenced by pathogen infections, including the combined effects of avian astrovirus and goose hemorrhagic polyomavirus [12]. Since 2017, severe and fatal visceral gout has spread among 5- to 20-day-old goslings in China. This disease is characterized by visceral uric acid deposition on the surface of the heart, liver, kidney, and joints. GAstV has been identified as the causative agent of this disease [3,13,14,15,16]. In recent years, cases of gout caused by a GAstV infection in goslings have continued to increase and have spread throughout most provinces in China, including Shandong, Henan, Hebei, Hunan, Anhui, Hubei, Jiangsu, Zhejiang, Guangdong, and Heilongjiang. In goslings, the incidence of GAstV is as high as 80%, with a staggering 15–30% mortality rate. The virus has caused considerable economic losses to the global goose industry, especially in China, where losses from GAstV have exceeded $10 billion [7,17,18]. There is still no effective prevention strategy against this virus. Continuous monitoring of the genetic diversity of dominant GAstV strains is particularly important for the development of targeted vaccines and appropriate drugs.

As a key region for the poultry industry in China, Hebei Province had no detailed reports on the epidemiology of GAstV before this study. Therefore, it is particularly important to conduct an in-depth investigation of the epidemiology of GAstV in Hebei Province. From January 2019 to December 2021, data from a total of 474 samples with a suspected clinical GAstV infection were collected from Hebei Province. Through detailed histological observation, pathological examination, virus isolation and identification, and genetic diversity analysis of these samples, we comprehensively revealed the distribution characteristics of GAstV in different periods and geographical regions, providing valuable scientific evidence for disease prevention and control. The results showed that GAstV-2 has become the main epidemic genotype, but GAstV-1 and mixed infections cannot be ignored and must be highly vigilant. Therefore, continuous and long-term monitoring work is crucial for understanding the dynamics of the epidemic situation and formulating effective prevention and control strategies. The results of this study increase the understanding of GAstV in Chinese waterfowl, provide important support for formulating scientific and reasonable prevention and control measures, and further promote the healthy development of China’s goose industry.

## 2. Materials and Methods

### 2.1. Clinical Cases and Sample Collection

From 2019 to 2021, a total of 474 suspected clinical cases of goose astrovirus infection were collected in Hebei Province, China, covering almost all municipalities in Hebei Province (Table 1). All geese were dissected and samples were collected in the biosafety laboratory. The heart, liver, spleen, lung, kidney, and muscle were collected from each goose. The samples were divided into two groups. One group was used for nucleic acid extraction. The other group was fixed with 10% neutral formalin and stained with hematoxylin-eosin (HE) and urate stain (GMS Method).

### 2.2. Virus Detection

To identify the virus in the samples, several common viruses in poultry were tested, including goose astrovirus 1 (GAstV-1), goose astrovirus 2 (GAstV-2), duck astrovirus (DAstV), goose coronavirus (GCoV), goose parvovirus (GPV), chicken astrovirus (CAstV), goose pegivirus (GPgV), goose circovirus (GoCV), avian influenza virus (AIV), goose hemorrhagic polyomavirus (GHPV), goose reovirus (GRV), duck tembusu virus (DTMUV), and Newcastle disease virus (NDV). Nucleic acids were extracted from clinical samples using the FastPure Viral DNA/RNA Mini Kit (RC311, Vazym, Nanjing, China). Samples that were only GAstV positive were used for subsequent studies. PCR or RT—PCR was performed as reported previously [19]. The primers used in this study are listed in Supplementary Table S1. All the primers used in the study were synthesized by Sangon Co., Ltd. (Shanghai, China).

### 2.3. Histopathological Examination

After PCR detection, samples positive for GAstV only were screened and fixed with 10% neutral formalin. After fixation, the samples were subjected to paraffin embedding. Subsequently, we cut the embedded sample into 4 μm slices for subsequent staining. For histological observation, a hematoxylin and eosin staining kit (C0105S, Beyotime, Haimen, China) was used for HE staining, and a urate stain kit (G3030, Solarbio, Beijing, China) was used for urate staining. The entire dyeing process was carried out in strict accordance with the operating instructions provided by the manufacturer. Finally, histopathological changes in the samples were observed under an optical microscope (BX53F, Olympus, Tokyo, Japan).

### 2.4. Virus Isolation and Identification

Only samples positive for GAstV were homogenized in sterile phosphate-buffered saline (PBS). Homogenized tissue samples were centrifuged at 8000× *g* at 4 °C for 20 min, and the supernatant was filtered through a 0.22 µm filter membrane (Millipore, Billerica, MA, USA). Finally, 200 µL of filtered supernatant was inoculated into the chorioallantoic cavity of 11-day-old goose embryos. The goose embryos used for GAstV isolation were purchased from a healthy farm in Hebei Province and were free of waterfowl viruses, including GAstV, GCoV, GPV, GPgV, GoCV, AIV, GHPV, GRV, TMUV, and DNV. Goose embryos were incubated at 37 °C and checked daily, and allantoic fluid from dead goose embryos 24 h later and viable goose embryos 7 days after inoculation were harvested under sterile conditions. Finally, the allantoic fluid was identified by RT—PCR or PCR, and allantoic fluid positive for GAstV only was stored at −80 °C for further study.

### 2.5. Whole-Genome Sequencing

Based on previous studies, extracted GAstV RNA was amplified by RT—PCR to obtain the full GAstV genome [19]. The GAstV whole-genome primers used are shown in Supplementary Table S2. The PCR products were subsequently sequenced by Shanghai Sangon Biotech Co., Ltd. (Shanghai, China) and submitted to the NCBI GenBank database (https://www.ncbi.nlm.nih.gov/genbank/, 6 March 2024).

### 2.6. Genetic Diversity Analysis

The BLAST program was used to perform a nucleotide sequence similarity search based on the GAstV whole-genome nucleotide sequence, ORF1a nucleotide-derived amino acid sequence, ORF1b nucleotide-derived amino acid sequence, and ORF2 nucleotide-derived amino acid sequence in the NCBI database. Phylogenetic analysis was performed with MEGA software, version 7.0 (Sunderland, MA, USA), employing the neighbor-joining (NJ) method with a 1000 bootstrap value. Information on all strains used in the genetic diversity analysis is provided in Supplementary Table S3.

## 3. Results

### 3.1. Clinical Symptoms

**Figure 1:**
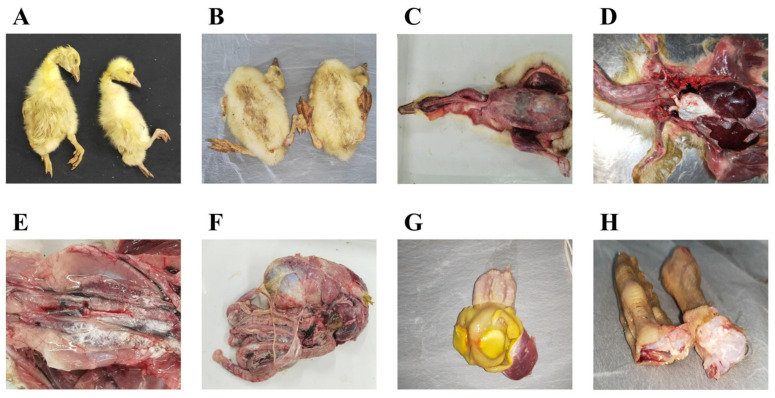
Clinical symptoms of suspected GAstV infection in geese. (**A**) Seven-day-old goslings suspected of having gout; (**B**) 15-day-old goslings suspected of having gout; (**C**) Urate deposited in the subcutaneous, tracheal serosa, pectoralis muscle, and leg muscle of geese; (**D**) Urate deposited in the heart, lung, and leg muscle of geese; (**E**) Urate deposited in the kidneys of geese; (**F**) Urate deposited in the splenic, glandular gastric, peritoneal gastric, and intestinal serosa of geese; (**G**) Ulcers with urate deposits were observed on the gastric mucosa of the gizzard; (**H**) Urate deposits were also found in the lumen of the tarsal joint.

The geese suspected of having a GAstV infection exhibited symptoms such as mental depression, loss of appetite for food, weight loss, difficulty walking, and white feces. Death occurs after the onset of these symptoms (Figure 1A,B), mostly in goslings aged 1–3 weeks. The necropsy results showed pathological changes typical of gout, with urate deposits in various joints and organs. A series of obvious pathological changes were found during the necropsy of the diseased geese. Obvious white urate deposits were observed subcutaneously on the serous surface of the trachea and on the surface of the pectoral and leg muscles (Figure 1C). Moreover, white urate deposits were also found in the pericardium (Figure 1D), liver serosa (Figure 1D), lungs (Figure 1D), kidneys (Figure 1E), splenic serosa (Figure 1F), adenogastric serosa (Figure 1F), myogastric serosa (Figure 1F), and intestinal serosa (Figure 1F). In addition, ulcers with urate deposits were observed on the gastric mucosa of the gizzard (Figure 1G). Urate deposits were also found in the lumen of the tarsal joint (Figure 1H). These findings further revealed the abnormal uric acid metabolism in diseased geese. Gastric mucosal ulceration and urate deposits were present in the gizzard (Figure 1G). Urate deposits were also visible in the tarsal joint cavity (Figure 1H). According to Table 1, among the clinical manifestations of suspected GAstV-infected geese, white feces (61.60%, 292/474) and mental depression (57.59%, 273/474) were the most common, followed by difficulty walking (57.17%, 271/474), no appetite for food (55.91%, 265/474), and weight loss (53.80%, 255/474). We also counted and analyzed the clinical deaths and found that 49.79% (236/474) of the deaths were associated with goose astrovirus infection. The mortality rate was 16.53% (39/236) in GAstV-1, 32.63% (77/236) in GAstV-2, and 50.85% (120/236) in mixed infections, and particularly, in young geese, the mortality rate was 41.03% (16/39) in GAstV-1, 54.55% (42/77) in GAstV-2, and 65.83% (79/120) in mixed infections.

### 3.2. Geographic and Temporal Distribution of Samples That Tested Positive for GAstV

**Figure 2:**
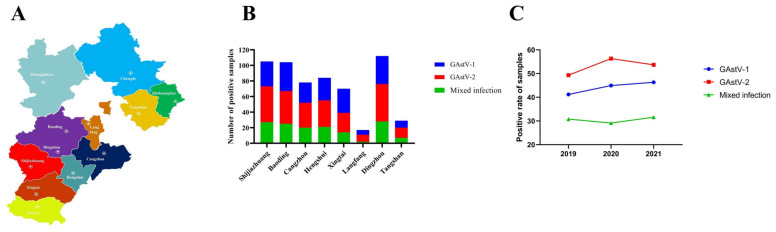
Detection results of GAstV for 474 samples. (**A**) Map of Hebei Province; (**B**) The number of positive samples of GAstV in different regions of Hebei Province; (**C**) Positivity of GAstV-positive samples in the different years.

From January 2019 to December 2021, a total of 474 suspected cases of GAstV infection were collected in Hebei Province. Of the 474 suspected cases of infection, 206 (43.46%) tested positive for GAstV-1, 249 (52.53%) tested positive for GAstV-2, and 144 (30.38%) tested positive for both GAstV-1 and GAstV-2 (Supplementary Table S4). The number of positive samples and the positivity rates for all regions during the study period are shown in Figure 2. In 2019, 41.18% (91/221) of all regions were positive for GAstV-1, 49.32% (109/221) of all regions were positive for GAstV-2, and 30.77% (68/221) of all regions were mixed positive. In 2020, the rate of GAstV-1 positivity in all regions was 44.94% (71/158), the rate of GAstV-2 positivity in all regions was 56.33% (89/158), and the rate of mixed positivity in all regions was 29.11% (46/158). In 2021, 46.32% (44/95) of all regions were positive for GAstV-1, 53.68% (51/95) were positive for GAstV-2, and 31.58% (30/95) were mixed positive.

### 3.3. Histopathological Changes

**Figure 3:**
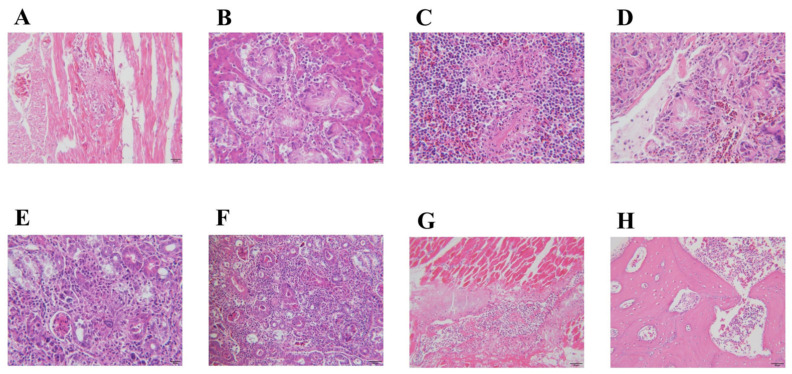
HE staining results. (**A**) Urate crystals were observed in myocardial fibers surrounded by monocytes; (**B**) Urate crystals were observed in the liver surrounded by epithelioid cells; (**C**) Urinate crystals were observed in the spleen surrounded by epithelioid cells and lymphocytes; (**D**) Urate crystals were observed in the lung surrounded by monocytes, epithelioid cells, and multinucleated giant cells; (**E**) Urate crystals were observed in the kidney surrounded by epithelioid cells; (**F**) Lymphocytes infiltrated in the renal interstitium were observed; (**G**) Coagulation necrosis of the leg muscles, precipitation of urate crystals, and infiltration of lymphocytes and monocytes were observed; (**H**) Urate crystals and infiltrated inflammatory cells were observed in the tarsal joint cavity. The scale bars are 50 μm in (**G**) and 20 μm in all other figures.

**Figure 4:**
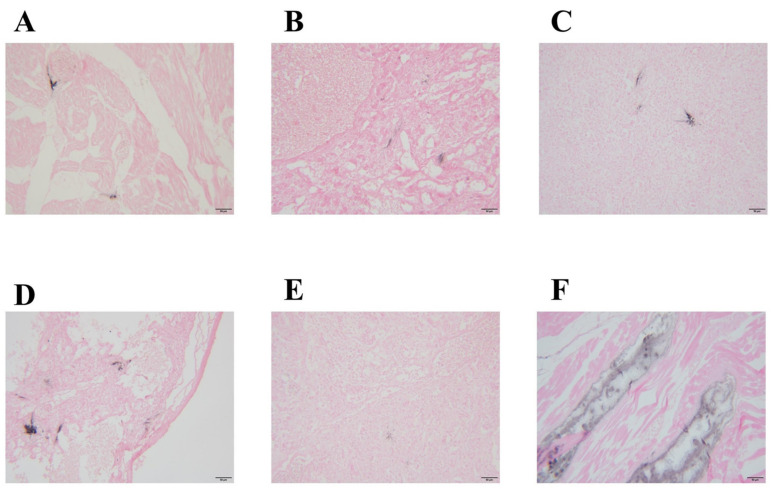
Results of urate staining. (**A**) myocardium; (**B**) liver; (**C**) spleen; (**D**) lung; (**E**) kidney; (**F**) leg muscle. The scale bar represents 50 μm.

Figure 3 shows the histopathological changes. Urate crystals are observed in the myocardium, surrounded by mononuclear cells, with infiltration of mononuclear cells between myocardial fibers and local myofibrillar rupture (Figure 3A). Urate crystals are also observed within the liver and are surrounded by epithelioid cells (Figure 3B). In the spleen, urate crystals are surrounded by epithelioid cells and lymphocytes (Figure 3C). The lungs showed urate crystals surrounded by mononuclear cells, epithelioid cells, and multinucleated giant cells (Figure 3D). The kidneys demonstrated urate crystals, with lymphocytic and mononuclear cell infiltration in the interstitial spaces of the local field of view (Figure 3E,F). Coagulation necrosis was observed in the local field of the leg muscles, accompanied by urate crystal deposition and lymphocytic and mononuclear cell infiltration (Figure 3G). Urate crystals and inflammatory cell infiltration were present in the tarsal joints (Figure 3H). Figure 4 shows the results of urate staining, revealing the presence of black urate crystals in the myocardium (Figure 4A), liver (Figure 4B), spleen (Figure 4C), lungs (Figure 4D), kidneys (Figure 4E), and leg muscles (Figure 4F).

### 3.4. Virus Isolation and Phylogenetic Trees

**Figure 5:**
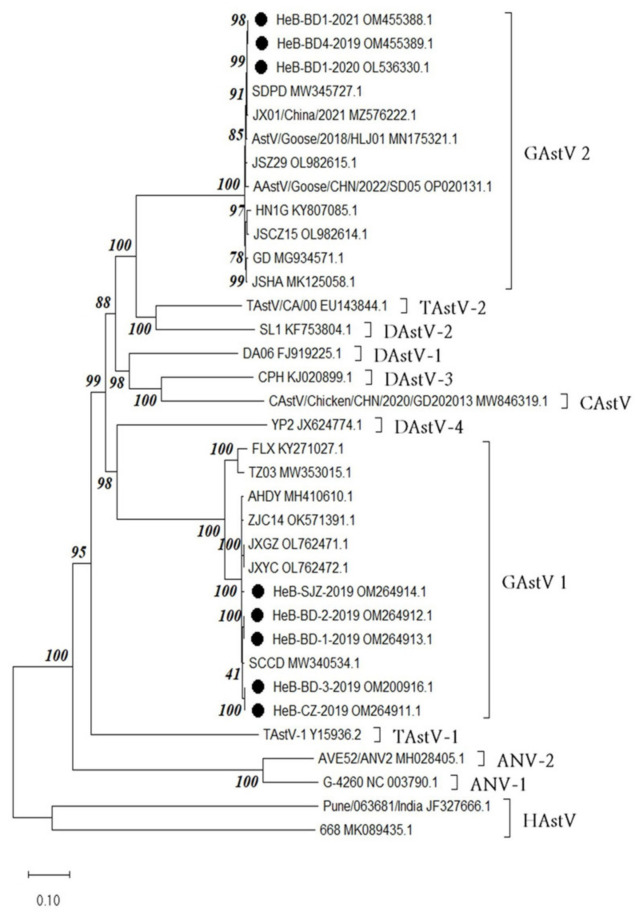
Phylogenetic tree based on the genome nucleotide sequence of GAstV.

**Figure 6:**
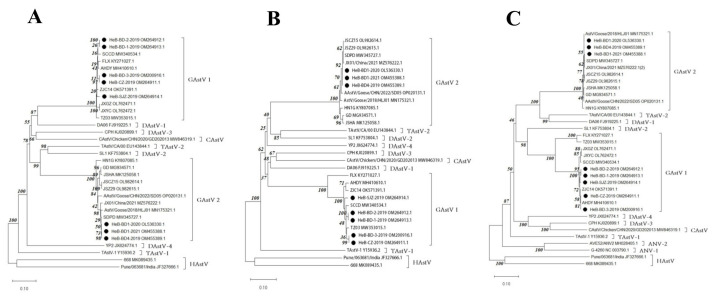
Phylogenetic trees of the GAstV gene based on its encoded amino acid sequences. (**A**) ORF1a; (**B**) ORF1b; (**C**) ORF2.

From 206 samples that tested positive for GAstV-1, five strains with different whole gene sequences were obtained, and all goose embryos inoculated with these five strains died within 84 h post-infection (hpi). Among the 249 samples that tested positive for GAstV-2, three strains with different full-length gene sequences were isolated, and all goose embryos inoculated with these three strains died within 48 h post-infection (hpi). The time to death after the inoculation of goose embryos with each strain is shown in Supplementary Table S5. Phylogenetic tree analysis revealed that the eight isolated GAstV strains belong to two major evolutionary branches (Figure 5 and Figure 6). Specifically, the HeB-CZ-2019, HeB-BD-2-2019, HeB-BD-1-2019, HeB-SJZ-2019, and HeB-BD-3-2019 strains clustered with the GAstV-1 reference strains in the same evolutionary branch, exhibiting a relatively long genetic distance from other avian astroviruses and the greatest genetic distance from human astroviruses, the sequence identity among the five strains was 98.9–99.9%. The HeB-BD1-2020, HeB-BD1-2021, and HeB-BD4-2019 strains grouped into a major branch with recently reported GAstV-2 reference strains, also showing a relatively long genetic distance from other avian astroviruses and the farthest genetic distance from human astroviruses, the sequence identity among the three strains was 99.6–99.8%.

## 4. Discussion

Since 2017, China’s goose farming industry has been severely impacted by severe visceral gout disease, and the sudden outbreak and widespread spread of this disease have caused heavy economic losses to the industry [7,9,13,20,21]. Worryingly, since the beginning of 2020, the epidemic has shown an increasing trend, with a fatality rate of more than 30%. Its main symptoms are visible urate deposits on internal surfaces such as the heart, liver, and kidneys, as well as in the joints [22]. Unfortunately, there are no effective prevention strategies for this virus. Continuous monitoring of the genetic diversity of the dominant GAstV strains is particularly important for developing targeted vaccines and appropriate drugs. Since Hebei Province is the main producing area of Chinese geese, this study carried out an epidemiological investigation of GAstV in Hebei Province for the first time and analyzed the characteristics of GAstV in different periods and regions. This not only helps to better understand the transmission law and pathogenic mechanism of the virus but also provides an important scientific basis for the prevention and control of the disease, which is highly important for ensuring the healthy development of the poultry industry and safeguarding the interests of farmers.

Based on clinical observations, the geese suspected of having a GAstV infection in this study exhibited symptoms such as white feces, mental depression, difficulty walking, loss of appetite, and weight loss, which are consistent with previous research reports [23]. Notably, white feces and mental depression were the most common symptoms observed in geese suspected of having a GAstV infection. Therefore, prompt detection should be conducted once these symptoms appear to confirm a GAstV infection in geese, allowing timely control measures and reduced losses in the poultry industry. Through HE staining analysis, typical pathological changes, including urate deposition and inflammatory cell infiltration, were observed in the myocardium, liver, and kidneys of GAstV-infected geese. Additionally, uric acid staining confirmed the widespread presence of uric acid in the myocardium, liver, spleen, lungs, kidneys, and leg muscles. This finding aligns with previous research reports, emphasizing that a GAstV infection can lead to urate deposition in multiple organs in geese, triggering gout [3,7,9,17]. The pathogenesis of GAstV-induced gout in goslings is primarily renal damage caused by uric acid excretion disorders or excessive uric acid production [1]. Therefore, a deeper understanding of this pathogenesis is crucial for the prevention and control of gout in goslings.

Previous studies have confirmed that the uric acid content in the serum of goslings infected with GAstV significantly increases, reaching a level of 3800 μmol/L on the 15th day post-infection [24]. Previous research revealed that the enzymatic activities and expression levels of xanthine oxidase (XOD) and adenosine deaminase (ADA) in the livers of goslings infected with GAstV are significantly elevated, showing a clear difference compared to those in the livers of uninfected control goslings. This increase in enzymatic activity directly leads to a significant surge in uric acid production in goslings, which is considered a key factor in the development of hyperuricemia and gout [25,26]. Notably, uric acid is primarily excreted through the kidneys, and during this process, organic anion transporters, multidrug resistance-associated protein 4 (MRP4), sodium-dependent phosphate transport protein 1, and sodium-potassium pumps play crucial roles, collectively forming the main mechanism of uric acid transport in poultry [27,28]. However, recent studies have further shown that a GAstV infection not only significantly reduces the mRNA expression level of MRP4 but also inhibits the activity of Na-K-ATP enzymes, directly leading to a decrease in the ability of the kidney to excrete uric acid [26]. More seriously, a GAstV infection can also cause kidney lesions, further compromising the excretory function of the kidney and exacerbating the occurrence of hyperuricemia and gout [29]. Therefore, the use of drugs that can protect the liver and kidneys in goslings infected with GAstV may play a crucial role. These findings undoubtedly provide important insights and directions for exploring effective treatment strategies against GAstV.

Research has revealed that GAstV possesses complex pathogenic ecology and genetic diversity. Due to its ability to transmit vertically and horizontally among goose embryos, goslings, and adult geese, the prevention and control of GAstV infections remain particularly challenging [3,30]. From January 2019 to December 2021, 474 cases of suspected GAstV infection in Hebei Province were included in this study. Among these suspected cases, 206 (43.46%) tested positive for GAstV-1, 249 (52.53%) tested positive for GAstV-2, and 144 (30.38%) tested positive for both GAstV-1 and GAstV-2. Notably, despite increasing attention given to GAstV-2 since early 2020, this study revealed that GAstV-1 infections remain severe, and mixed infections are also a serious concern. Interestingly, over 65% of the mortality cases among goslings involved mixed infections. There were significant differences in the positive rates of GAstV among the different regions in Hebei Province, which may be related to differences in farming practices, stock numbers, and trade patterns. Regions with large stock numbers, extensive farming methods, and casual live-bird trading tend to have higher positive rates. Hebei Province, as a major waterfowl breeding province in China, produces more than one hundred million waterfowl annually. However, due to relatively backward farming techniques, inadequate biosecurity measures, and the continuous growth of waterfowl numbers, epidemic outbreaks are frequent in Hebei. Additionally, the close geographical proximity and convenient transportation among cities in Hebei, along with frequent goose-product trading, facilitate the potential spread of goose viruses across multiple regions. To effectively block the transmission of GAstV across different regions, it is crucial to strictly limit cross-regional trade in live waterfowl and goose embryos. It is necessary to rely on precise detection methods, combined with strict quarantine measures and disinfection practices, to build an effective barrier against the further spread of the virus. This not only poses a challenge to the waterfowl breeding industry in Hebei Province but also represents a severe test for the entire poultry industry and public health safety. Therefore, it is imperative to promote the prevention and control of GAstV by adopting practical and effective measures to ensure the healthy development of the waterfowl breeding industry and public health safety.

Previous studies have shown that the mortality rate of goose embryos inoculated with GAstV is as high as 100%. This infection not only severely hinders embryonic development, leading to a significant decline in hatchability, but also causes severe hemorrhage in goose embryos [24]. In this study, we successfully isolated eight GAstV strains from clinical samples and infected goose embryos and observed symptoms similar to those reported in previous research. Genetic diversity analysis based on the full-genome nucleotide sequence of GAstV, as well as the amino acid sequences derived from the nucleotide sequences of ORF1a, ORF1b, and ORF2, revealed that GAstV can be classified into two distinct branches: GAstV-1 and GAstV-2. Specifically, the strains isolated in this study, including HeB-CZ-2019, HeB-BD-2-2019, HeB-BD-1-2019, HeB-SJZ-2019, and HeB-BD-3-2019, belong to GAstV-1, while HeB-BD1-2020, HeB-BD1-2021, and HeB-BD4-2019 belong to GAstV-2. Furthermore, genetic-diversity analysis revealed that the strains isolated in this study have a relatively distant genetic relationship with other avian astroviruses and an even more distant relationship with human astroviruses. These findings further confirm the complex pathogenic ecology and genetic diversity of GAstV, suggesting that further experimental studies are needed to investigate the biological differences among these isolated strains. With the prevalence of GAstV in the goose-breeding industry, it is foreseeable that this disease will continue to pose a serious threat to the healthy development of the goose-breeding industry in Hebei Province. In addition, our study has several limitations. As there are no commercialized antibody detection kits for GAstV, we were unable to perform serology studies. Moreover, in terms of genetic evolutionary analyses, we will use appropriate programs for phylodynamic and temporal analyses in future research.

## 5. Conclusions

In conclusion, this is the first epidemiological investigation of GAstV conducted in Hebei Province, China, which revealed the prevalence trend and potential risks of GAstV in the province. These results indicate that GAstV-2 has become the primary prevalent genotype, while GAstV-1 and mixed infections still cannot be ignored and should receive increased attention. Therefore, continuous and long-term monitoring efforts are crucial. In addition, this study revealed that GAstV-2 was stronger than GAstV-1 in terms of pathogenicity, clinical symptoms, morbidity, and mortality, but the effects of mixed infections on these aspects are not clear, and more trials are needed to investigate this topic in the future. This research not only deepens our understanding of GAstV in Chinese waterfowl but also provides a scientific basis for developing effective prevention and control measures. This will promote the healthy development of the goose industry in China and ensure the sustained stability of the waterfowl industry. We look forward to continuing further research in the future, exploring more prevention and control strategies, and contributing more to the long-term development of the goose industry.

## Figures and Tables

**Table 1:** Information on the clinical cases in this study.

Origin City	Numbers	Clinical Symptoms				
		Mental Depression *	No Appetite for Food	Weight Loss	Difficulty Walking	White Feces
Shijiazhuang	73	41	32	15	52	36
Baoding	85	32	67	43	37	53
Cangzhou	66	43	26	29	41	29
Hengshui	64	35	42	36	28	37
Xingtai	51	26	29	37	21	38
Langfang	29	19	11	17	21	19
Dingzhou	80	65	39	67	57	69
Tangshan	26	12	19	11	14	11
Total numbers	474	273	265	255	271	292

* Mental depression performance: Activity reduction (geese become inactive, exhibit slow action, and take a long time to keep still); loose feathers (goose feathers become fluffy and messy, lose the original luster and neatness); slow reaction (for external stimuli, the reaction of the goose becomes slow).

## Data Availability

The original contributions presented in the study are included in the article, further inquiries can be directed to the corresponding authors.

## Supplementary Materials

The following supporting information can be downloaded at: https://www.mdpi.com/article/10.3390/microorganisms12050990/s1, Table S1: Primers used for the detection of goose viruses in this study; Table S2: Primers used to amplify the complete genome of 2 genotypes GAstV; Table S3: Basic information about related AstVs; Table S4: The detection results of GAstV for 474 samples; Table S5: Time of death of goose embryos after AstVs inoculation.
