# Supplementary material for: Epidemiological Investigation of Goose Astrovirus in Hebei Province, China, 2019–2021
Source: Microorganisms. 2024 May 14;12(5):990. doi: 10.3390/microorganisms12050990 (PMC11123679; doi:10.3390/microorganisms12050990)
Supplement: Supplementary file 1 [file microorganisms-12-00990-s001.zip › Supplementary Materials.pdf]

Table S1. Primers used for the detection of goose viruses in this study

Primers	Primer sequence	Fragment size
G1-ORF1b-F	CTGGACAGGCGAATTAGAAG	
G1-ORF1b-R	CCAAAGACATCGGCATACA	482
G2-ORF1b-F	GCCTCTCTTCTGGCGGATAC	
G2-ORF1b-	CCCTGAGTAACCTGAGCGTC	284
DAstV F	ANRTYTTTGGNATGTGGG	
DAstV R	GAAGTCGGSSCCRACTTC	301
GCoV F	ACTCARWTRAATYTNAAATAYGC	
GCoV R	TCACAYTTWGGATARTCCCA	251
GPV F	CCAAGCTACAACAACCACAT	
GPV R	TGAGCGAACATGCTATGGAAGG	539
CAstVF	GAYCARCGAATGCGRAGRTTG	
CAstVR	TCAGTGGAAGTGGGKARTCTAC	362
GPgV F	CGACAAGGGTGCCTGCTGA	
GPgV R	CCGTAGGTCGCATAGGT	194
GoCV F	CGATTAATAACCCTACCTTTGA	
GoCV R	GACCAATCAGAACGATGACC	462
AIV F	TTCTAACCGAGGTCGAAAC	
AIV R	AAGCGTCTACGCTGCAGTCC	229
GHPV F	GGTTAATTCCCTGACTCACA	
GHPV R	AATAAGCTTGCAGTTTCAGC	1270
GRV F	CTTTTTGAGTCCTTGTGCAGCCATG	
GRV R	GTAAGAGTCCAAGTCGTGGCAGAG	1185
DTMUV F	GCCACGGAATTAGCGGTTGT	
DTMUV R	TAATCCTCCATCTCAGCGGTGTAG	401
NDV F	TGGGCTCCAGACCTTTTAC	
NDV	TGTTGGCAGCATTTTGTTT	451

Table S2. Primers used to amplify the complete genome of 2 genotypes GAstV

Primer name	Primer sequence（5′→ 3′）	Segment position	Fragment size
1F-1	CCGAAAGCGTTGGTGAGAG	1-19	1553
1R-1	GGGTCCCACAGCTCATTGA	1535-1553	
1F-2	GTACCACTTCAGATCTTACTG	1499-1519	1621
1R-2	GGTTCATGCACCAGTTACAT	3100-3119	
1F-3	CCTTGGAGCAGAGGAAGA	3081-3098	1479
1R-3	GACAAGCCTTCAGGTACC	4542-4559	
1F-4	AAGGTGCAGATGGTACCTG	4531-4549	1427
1R-4	ATTTGACCCTGCAGCTCC	5940-5957	
1F-5	GGAAGATCTTTGGAGCTGC	5929-5947	1327
1R-5	TTGGTTCAAAAACAGAACCG	7236-7255	
2F-1	GCATGGGGAAACAGCGATA	1-19	1561
2R-1	TCGACACAGTACCGTCAGG	1543-1561	
2F-2	GCGTGTTCCACTATTACAGTC	1516-1536	1571
2R-2	CTCGACCCACACCACTTACA	3067-3086	
2F-3	TCAACCTCTTGAGCAGCGC	3039-3057	1724
2R-3	TCACTGGCAGCGTGCTCCT	4744-4762	
2F-4	ATCAACACGGTTGAGGAGC	4731-4749	1564
2R-4	CCCACCAATTGTGTGTTCC	6276-6294	
2F-5	CGTGGAAGGACTTCAACATC	6246-6265	871
2R-5	TAAACAAAAACCCGGTCAGG	7097-7116	
2F-6	CATGAGTGATCTCTTCACTAGCAGC	6939-6963	241
2R-6	TTTTTTTTTTTTTTTTTTTTATAAGATTTTAAATGC	7139-7175	

Table S3. Basic information about related AstVs

Strain name	Genome accession number	Genotype
**HeB-CZ-2019***	OM264911.1	GAstV 1
**HeB-BD-2-2019***	OM264912.1	GAstV 1
**HeB-BD-1-2019***	OM264913.1	GAstV 1
**HeB-SJZ-2019***	OM264914.1	GAstV 1
**HeB-BD-3-2019***	OM200916.1	GAstV 1
JXGZ	OL762471.1	GAstV 1
JXYC	OL762472.1	GAstV 1
ZJC14	OK571391.1	GAstV 1
SCCD	MW340534.1	GAstV 1
TZ03	MW353015.1	GAstV 1
AHDY	MH410610.1	GAstV 1
FLX	KY271027.1	GAstV 1
**HeB-BD1-2020***	OL536330.1	GAstV2
**HeB-BD1-2021***	OM455388.1	GAstV2
**HeB-BD4-2019***	OM455389.1	GAstV2
AAstV/Goose/CHN/2022/SD05	OP020131.1	GAstV2
JX01/China/2021	MZ576222.1	GAstV2
JSZ29	OL982615.1	GAstV2
SDPD	MW345727.1	GAstV2
AstV/Goose/2018/HLJ01	MN175321.1	GAstV2
GD	MG934571.1	GAstV2
JSHA	MK125058.1	GAstV2
JSCZ15	OL982614.1	GAstV2
HN1G	KY807085.1	GAstV2
DA06	FJ919225.1	DAstV-1
SL1	KF753804.1	DAstV-2
CPH	KJ020899.1	DAstV-3
YP2	JX624774.1	DAstV-4
CAstV/Chicken/CHN/2020/GD202013	MW846319.1	CAstV
G-4260	NC_003790.1	ANV-1
AVE52-ANV2	MH028405.1	ANV-2
TAstV-CA-00	EU143844.1	TAstV-2
TAstV-1	Y15936.2	TAstV-1
Pune-063681-India	JF327666.1	HAstV
668	MK089435.1	HAstV

Note: * represents the strain isolated in this study.

Table S4. The detection results of GAstV for 474 samples

Origin city	Sample time	Sample quantity	Number of genotype 1 positive	Number of genotype 2 positive	Mixed positive number
Shijiazhuang	2019	34	41.18%(14/34)	61.67%(21/34)	38.24%(13/34)
	2020	25	48%(12/25)	60%(15/25)	36%(9/25)
	2021	14	42.86%(6/14)	71.43%(10/14)	35.71%(5/14)
	Total	73	43.84%(32/73)	63.01%(46/73)	36.99%(27/73)
Baoding	2019	38	39.47%(15/38)	44.74%(17/38)	31.58%(12/38)
	2020	27	44.44%(12/27)	48.15%(13/27)	29.63%(8/27)
	2021	20	50%(10/20)	60%(12/20)	25%(5/20)
	Total	85	43.53%(37/85)	49.41%(42/85)	29.41%(25/85)
Cangzhou	2019	31	38.71%(12/31)	48.39%(15/31)	25.81%(8/31)
	2020	23	34.78%(8/23)	39.13%(9/23)	26.09%(6/23)
	2021	12	50%(6/12)	66.67%(8/12)	50%(6/12)
	Total	66	39.39%(26/66)	48.48%(32/66)	30.30%(20/66)
Hengshui	2019	28	42.86%(12/28)	53.57%(15/28)	35.71%(10/28)
	2020	20	45%(9/20)	75%(15/20)	35%(7/20)
	2021	16	50%(8/16)	25%(4/16)	25%(4/16)
	Total	64	45.31%(29/64)	53.13%(34/64)	32.81%(21/64)
Xingtai	2019	24	66.67%(16/24)	58.33%(14/24)	37.50%(9/24)
	2020	18	66.67%(12/18)	44.44%(8/18)	16.67%(3/18)
	2021	9	33.33%(3/9)	33.33%(3/9)	22.22%(2/9)
	Total	51	60.78%(31/51)	49.02%(25/51)	27.45%(14/51)
Langfang	2019	13	15.38%(2/13)	23.08%(3/13)	7.69%(1/13)
	2020	9	22.22%(2/9)	44.44%(4/9)	11.11%(1/9)
	2021	7	28.57%(2/7)	28.57%(2/7)	0(0/7)
	Total	29	20.69%(6/29)	31.03%(9/29)	6.90%(2/29)
Dingzhou	2019	39	41.03%(16/39)	46.15%(18/39)	30.77%(12/39)
	2020	27	44.44%(12/27)	74.07%(20/27)	33.33%(9/27)
	2021	14	57.14%(8/14)	71.43%(10/14)	50%(7/14)
	Total	80	45%(36/80)	60%(48/80)	35%(28/80)
Tangshan	2019	14	28.57%(4/14)	42.86%(6/14)	21.43%(3/14)
	2020	9	44.44%(4/9)	55.56%(5/9)	33.33%(3/9)
	2021	3	33.33%(1/3)	66.67%(2/3)	33.33%(1/3)
	Total	26	34.62%(9/26)	50%(13/26)	26.92%(7/26)
Total	-	474	43.46%(206/474)	52.53%(249/474)	30.38%(144/474)

Table S5. Time of death of goose embryos after AstVs inoculation (n=10)

Strain name	Time*
HeB-CZ-2019	52.20±12.35
HeB-BD-2-2019	79.20±6.81
HeB-BD-1-2019	80.40±3.10
HeB-SJZ-2019	73.20±10.12
HeB-BD-3-2019	75.60±6.45
HeB-BD1-2020	43.20±5.98
HeB-BD1-2021	30.00±4.90
HeB-BD4-2019	40.00±8.59

*hours post infection (hpi)
